# Current Taxonomical Situation of *Streptococcus suis*

**DOI:** 10.3390/pathogens5030045

**Published:** 2016-06-24

**Authors:** Masatoshi Okura, Makoto Osaki, Ryohei Nomoto, Sakura Arai, Ro Osawa, Tsutomu Sekizaki, Daisuke Takamatsu

**Affiliations:** 1 Division of Bacterial and Parasitic Diseases, National Institute of Animal Health, National Agriculture and Food Research Organization, 3-1-5 Kannondai, Tsukuba, Ibaraki 305-0856, Japan; mokura@affrc.go.jp (M.O.); osaki@affrc.go.jp (M.Os.); 2 Department of Infectious Diseases, Kobe Institute of Health, 4-6-5 Minatojima-Nakamachi, Chuo-ku, Kobe, Hyogo 650-0045, Japan; ryohei_nomoto@office.city.kobe.lg.jp; 3 Research Center for Food Safety, Graduate School of Agricultural and Life Sciences, The University of Tokyo, 1-1-1 Yayoi, Bunkyo-ku, Tokyo 113-8657, Japan; sakurairo1218@yahoo.co.jp (S.A.); asekizak@mail.ecc.u-tokyo.ac.jp (T.S.); 4 Organization for Advanced Science and Technology, Kobe University, 1-1 Rokko-dai, Nada-ku, Kobe, Hyogo 657-8501, Japan; tamie@opal.kobe-u.ac.jp; 5 The United Graduate School of Veterinary Sciences, Gifu University, 1-1 Yanagido, Gifu, Gifu 501-1193, Japan

**Keywords:** *Streptococcus suis*, taxonomic analyses, species demarcation, *S. suis*-like strains

## Abstract

*Streptococcus suis*, a major porcine pathogen and an important zoonotic agent, is considered to be composed of phenotypically and genetically diverse strains. However, recent studies reported several “*S. suis*-like strains” that were identified as *S. suis* by commonly used methods for the identification of this bacterium, but were regarded as distinct species from *S. suis* according to the standards of several taxonomic analyses. Furthermore, it has been suggested that some *S. suis*-like strains can be assigned to several novel species. In this review, we discuss the current taxonomical situation of *S. suis* with a focus on (1) the classification history of the taxon of *S. suis*; (2) *S. suis*-like strains revealed by taxonomic analyses; (3) methods for detecting and identifying this species, including a novel method that can distinguish *S. suis* isolates from *S. suis*-like strains; and (4) current topics on the reclassification of *S. suis*-like strains.

## 1. Introduction

*Streptococcus suis* is an important swine pathogen responsible for severe economic loss to the global swine industry [1,2,3]. *S. suis* can cause a variety of diseases, including meningitis, sepsis, endocarditis, arthritis, and pneumonia [1,2,3], while healthy pigs frequently carry this bacterium, particularly in their upper respiratory tracts and tonsils [4]. *S. suis* is also recognized as an emerging zoonotic pathogen that can be transmitted to humans from infected pigs or contaminated raw pork products [1,2,3,5]. In addition to pigs and humans, *S. suis* infection sporadically occurs in other animals, such as cattle, sheep, goats, boars, horses, cats, dogs, and birds [1,6,7].

*S. suis* strains were serologically classified on the basis of the different antigenicity of their capsular polysaccharides (CPSs), and 35 serotypes (serotypes 1–34 and serotype 1/2 that reacts with both serotypes 1 and 2 antisera) have been reported [8,9,10,11,12,13]. In addition, the new serotype Chz was recently proposed [14]. Serotyping of *S. suis* is mainly performed for the identification and diagnosis of clinical isolates. Most *S. suis* isolates from diseased pigs belong to a limited number of serotypes, including serotypes 2, 3, 7, and 9; however, the distribution of serotypes from clinical cases differs depending on the geographic location (e.g., serotypes 2 and 3 are the most prevalent serotypes in Canada and the United States, while serotype 9 is the most frequently found in some European countries [2]). In humans, most clinical cases were associated with serotype 2 strains [2]. On the contrary, isolates from healthy pigs and other animals were usually classified into more diversified serotypes, and serologically untypable strains were also frequently found in these animals ([2,15,16] and unpublished observation), implying that more serotypes are present in *S. suis* than those reported to date. *S. suis* strains have been genotyped into many different sequence types (STs) by multi-locus sequence typing (MLST), which is used in many laboratories globally to genotype this species [2,17]. As of April 2016, more than 700 STs were known in *S. suis* (MLST datasets are available from PubMLST [18]). These previous studies indicate that phenotypically and genotypically diverse strains are included in the taxon *S. suis*. However, the presence of several “*S. suis*-like strains” has recently begun to be reported. These strains had been identified as *S. suis* by the commonly used identification methods for this species, but they were regarded as non-*S. suis* strains when reverified by several taxonomic analyses [19,20,21]. That is, demarcation of the species *S. suis* is currently becoming obscure. In this review, to help understand the current taxonomical situation of *S. suis*, we discuss previous studies on this species with a focus on (1) the taxonomic classification history of *S. suis*; (2) *S. suis*-like strains identified by taxonomic analyses; (3) methods for the detection and identification of *S. suis*; and (4) current topics on the reclassification of *S. suis*-like strains.

## 2. Taxonomic and Serological Classification Histories of *S. suis*

**Figure 1:**
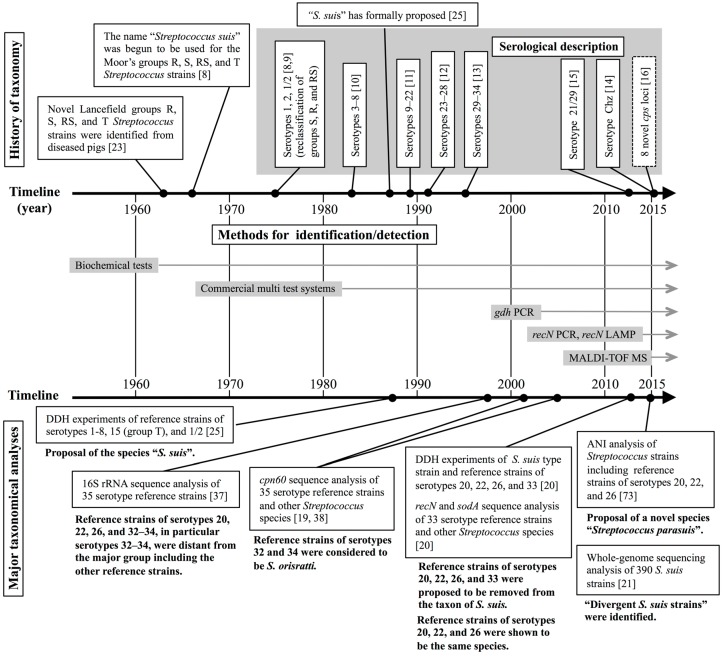
Timeline summary on the history of the taxon, serological description, identification/detection methods, and major taxonomical analyses (including the findings) of *S. suis*. DDH, DNA-DNA hybridization. ANI, average nucleotide identity.

Since the 1930s, streptococci have been classified by Lancefield grouping, which is based on the carbohydrate composition of bacterial cell wall antigens [22]. In the early 1960s, De Moor [23] assigned the *Streptococcus* strains from outbreaks and sporadic cases of septicemic infection in pigs into Lancefield groups designated R, S, RS, and T. In 1966, Elliott [8] revealed that Moor’s groups R and S were subgroups of Lancefield group D and regarded the strains of these groups as the new species “*Streptococcus suis*”. Furthermore, it was demonstrated that the major antigens of Moor’s groups R and S originated from their CPSs rather than their cell wall materials [8,9]. Then, Moor’s groups S, R, and RS streptococci were reclassified as *S. suis* serotypes 1, 2, and 1/2, respectively [8,9]. In 1983, six new serotypes (serotypes 3–8) were described by Perch et al. [10]. However, at that time, the name *S. suis* had not yet been included in the Approved Lists of Bacterial Names [24]. The formal proposal of the name “*S. suis*” was finally made in 1987 by Kilpper-Balz and Schleifer [25], and then, 26 additional serotypes (serotypes 9–34) were described for this species during the period from 1989 to 1995 [11,12,13]. In 2013, isolation of several *S. suis* serotype 21/29 strains from healthy pigs was reported [15]. Furthermore, in 2015, Pan et al. identified the novel serotype Chz in *S. suis* isolates from pigs with meningitis [14]. These taxonomical and serological classification histories of *S. suis* are summarized in Figure 1.

## 3. Several Taxonomic Analyses Revealed that Six *S. suis* Serotype Reference Strains are not *S. suis*

### 3.1. Taxonomic Standards for Species Delineation and Taxonomic Approaches for Phylogenetic Relationships in Bacteria

In bacteria, a DNA–DNA hybridization (DDH) similarity of ≥70% is the gold standard for assigning two strains to the same species [26,27]. However, researchers are hesitant to use DDH experiments due to the complex and time-consuming nature of the technique [28,29,30]. In the 1990s, the cost, technology, and methodologies of DNA sequencing improved dramatically, and many centers then possessed DNA sequencers. Subsequently, many taxonomic and phylogenetic studies have been conducted on the basis of the sequences of specific housekeeping genes and/or other polyphasic data including biochemical characteristics (reviewed in [31]).

In 1994, Stackebrandt and Goebel [32] suggested that sequence analysis of the 16S rRNA gene is potentially useful for the definition of a species in bacteria. The accumulated data on 16S rRNA gene sequences revealed that the correlation between the 16S rRNA sequence similarities and DDH values obtained for the same strain pairs is not linear [32,33]. However, in the dataset analyzed to date, it has been demonstrated that, below a threshold value of 97% 16S rRNA sequence similarity, the corresponding DDH values were always lower than 70% [32,33,34]. Therefore, it is now generally accepted that two strains are regarded as distinct species when the 16S rRNA sequence similarity between them is less than 97% [27,32], although higher threshold values (98.7%–99.0% or 98.2%–99.0%) have been recommended in several studies [33,34].

Sequencing analyses based on other housekeeping genes were also utilized for the discrimination of bacterial species because of the greater discriminating power than that of the 16S rRNA gene [31]. In *Streptococcus* species, the sequences of *sodA*, encoding the manganese-dependent superoxide dismutase, and *recN*, encoding a recombination/repair protein, displayed low similarity values at the species level and a high divergence value at the subspecies level relative to those of other housekeeping genes [35]. Furthermore, the minimal interspecies divergence in the sequences of *cpn60* (*groEL*), encoding the 60-kDa heat shock protein, was higher than those of other housekeeping genes in the *Streptococcus* species analyzed [36]. These previous studies suggested that sequence comparisons of *cpn60*, *sodA*, and *recN* are useful for identifying *Streptococcus* species and subspecies and conducting phylogenetic analysis.

### 3.2. Taxonomic Studies Using S. suis Serotype Reference Strains

DDH experiments on *S. suis* were conducted using 16 *Streptococcus* strains including 10 *S. suis* reference strains (serotypes 1–8, 1/2, and an original Moor’s group T strain that is currently assigned as the serotype 15 reference strain) for the formal proposal of the species “*S. suis*” [25]. All analyzed *S. suis* strains were confirmed to be the same species according to the DDH values (more than 80%) [25]. However, until a study in 2013 [20] (see below), no additional DDH data on *S. suis* strains had been reported, and reference strains of novel serotypes described in the interim (serotypes 9–14 and 16–34) were identified as *S. suis* on the basis of the biochemical characteristics of the strains [11,12,13].

In 1998, Chatellier et al. [37] reported a 16S rRNA sequencing analysis of 35 *S. suis* serotype reference strains (serotypes 1–34 and 1/2). In their data, the reference strains of serotypes 20, 22, 26, and 32–34 were located distant from the other 29 reference strains on the 16S rRNA-based phylogenetic tree [37]. In addition, the reference strains of serotypes 20, 22, 26, and 32–34 exhibited 16S rRNA sequence similarity values with the other 29 reference strains of less than 97% (serotypes 32–34) or 96.76%–98.27% (serotypes 20, 22, and 26) [37]. According to the generally accepted or recommended taxonomic criteria of 16S rRNA sequence similarity, these six serotype reference strains are suggested to be distinct species from *S. suis*. Indeed, Chatellier et al. demonstrated that on the 16S rRNA-based phylogenetic tree, the serotype 33 reference strain was more related to *Streptococcus acidominimus* than to the major group of *S. suis* isolates and that serotype 32 and 34 reference strains were more closely related to the pyogenic group of *Streptococcus*, which includes *Streptococcus agalactiae*, *Streptococcus parauberis*, *Streptococcus porcinus*, and *Streptococcus uberis* [37].

The *S. suis* reference strains of serotypes 20, 22, 26, and 32–34 were also separated from the other 29 reference strains via phylogenetic analysis based on the *cpn60* sequence [19,38]. In *Streptococcus* species, phylogenies inferred from *cpn60* sequence comparisons were found to be more discriminative than those inferred from 16S rRNA gene sequence comparisons [36]. Within *S. suis*, the *cpn60* sequences also displayed a higher level of diversity among the serotype reference strains than the 16S rRNA sequences [38]. Nevertheless, the *cpn60* sequences of serotype 32 and 34 reference strains shared more than 99% nucleotide identity with that of the *Streptococcus orisratti* type strain [19]. In contrast, the identities of the *cpn60* sequences between *S. orisratti* and the other *S. suis* strains included in the study were only 78%–79% [19]. Taking these results into account, *S. suis* reference strains of serotypes 32 and 34 are currently considered to be *S. orisratti* [19].

In 2013, Tien et al. [20] demonstrated that the *S. suis* reference strains of serotypes 20, 22, 26, and 33 were clearly distinguished from the other 29 serotype reference strains (serotypes 1–19, 21, 23–25, 27–31, and 1/2) by phylogenetic analyses using *sodA* and *recN* sequences. In addition, these four reference strains exhibited DDH values of less than 70% with the *S. suis* type strain (13.96%–33.87%) [20]. From these findings, the authors proposed that the serotype 20, 22, 26, and 33 strains should be removed from the taxon of *S. suis* [20].

The aforementioned findings on the taxonomic positions of the serotype 20, 22, 26, and 32–34 reference strains are summarized in Figure 1. These findings suggest that some non-*S. suis* strains may be included with the isolates identified as “*S. suis*” on the basis of their biochemical characteristics. Such non-*S. suis* strains (i.e., strains which were previously identified as *S. suis* but which are currently considered not to be *S. suis*) are referred to as “*S. suis*-like strains” throughout this review, for convenience. Precise identification of *S. suis* and *S. suis*-like strains may help us understand the epidemiology of this important zoonotic disease more accuracy; however, it is difficult to discriminate *S. suis*-like strains from “authentic *S. suis*” by commonly used routine methods for the identification of *S. suis*. To solve this problem, novel identification and detection methods for *S. suis* have recently been developed. In the next section, we summarize those novel methods as well as the standard methods, which have been used in the majority of laboratories for many years in the identification and detection of this species.

## 4. Methods for Identifying and Detecting *S. suis*

A timeline summary of the history of the methods for the identification/detection of *S. suis* is shown in Figure 1.

### 4.1. Routine Methods for Identifying and Detecting *S. suis*

*S. suis* is a gram-positive coccus arranged in pairs, short chains, or single [39,40]. On bovine or sheep blood agar plates, most *S. suis* strains are alpha-hemolytic after 24 h of incubation at 37 °C [39,40]. Alpha-hemolytic and gram-positive coccus isolates can be presumptively identified as *S. suis* by four tests: no growth in 6.5% NaCl agar, a negative Voges–Proskauer test, and the production of acid from either trehalose or salicin [2,39,40]. For more precise identification, serotyping is conducted after the following biochemical tests: arginine dihydrolase (positive), production of acid from lactose, sucrose, and inulin (positive), and production of acid from glycerol, mannitol, and sorbitol (negative) [2,40]. Commercial API^®^ multitest systems can be also used for the identification of *S. suis* [41,42,43], but these tests sometimes misidentify the isolates [2,3]. In clinical cases in pigs with typical clinical symptoms of *S. suis* infection, it is relatively easy to identify *S. suis* using the aforementioned tests [2]. By contrast, in human cases, misidentification can occur due to a lack of cognizance of this pathogen [2]. It is also difficult to identify the isolates from clinically healthy pigs or other animals using the aforementioned biochemical properties because strains of other *Streptococcus* species that are phenotypically similar to *S. suis* can be recovered from the same sites [2,3].

During the last decade, molecular biological approaches have been developed for detecting and identifying *S. suis* strains. One of the most widely used methods is a polymerase chain reaction (PCR) assay targeting the *S. suis*-distinctive sequences of the housekeeping gene *gdh*, encoding the glutamate dehydrogenase [44]. However, it was reported that certain *S. suis* isolates were not correctly identified as *S. suis* by this PCR [45]. In addition, isolates of other *Streptococcus* species (such as *Streptococcus gallolyticus*, *Streptococcus gallinaceus*, and *Streptococcus ovis*) could be misidentified as *S. suis* by this method [46,47]. One possible reason for these misidentifications is the design concept of the primers for the PCR. This *gdh* PCR assay was originally developed to detect all 35 serotype reference strains (serotypes 1–34 and 1/2) [44]. When the *gdh* PCR was developed, this design concept was reasonable because no strong evidence had been reported to reclassify some of the reference strains into other species. However, as described in an aforementioned section (Section 3.2), the *S. suis* serotype 20, 22, 26, and 32–34 reference strains are currently considered to be distinct species from *S. suis*; that is, although this PCR system was extremely useful, it does not match the current taxonomical situation of *S. suis*.

Serotyping of *S. suis* is useful for identifying clinical isolates because the method will provide further confirmation of the pathogen’s identity [2]. In particular, the detection of serotype 2 isolates is very important for diagnosing *S. suis* infection in humans. However, in *S. suis*, serotyping with all typing antisera is time-consuming, and preparing the antisera is not easy due to the high cost and labor associated with its production [48]. To solve these problems, several molecular biological approaches have been developed as practical and easy methods to aid in the serotyping of *S. suis* ([14,15,48,49,50,51,52,53,54,55,56,57,58,59,60] Summarized in Table 1). Some of these methods can discriminate almost all serotypes and be used as molecular serotyping methods (Table 1).

Matrix-assisted laser desorption ionization time-of-flight mass spectrum (MALDI-TOF MS) has recently emerged as a reliable high-throughput tool for the microbiological identification of clinical isolates [61]. MALDI-TOF MS-based identification has been reported for several *Streptococcus* species [62,63,64,65,66]. Recently, Pérez-Sancho et al. [67] reported the excellent performance of MALDI-TOF MS for the identification of *S. suis*. In their data, 96.9% of the tested *S. suis* isolates (125/129 isolates) were correctly identified using the *S. suis* MALDI Biotyper database updated with the spectra of three additional clinical isolates of serotypes 2, 7, and 9 [67]. However, because bacterial isolates identified as *S. suis* by *gdh* PCR [44] were used for evaluating the accuracy of MALDI-TOF MS for identifying *S. suis* in the study, *S. suis*-like strains might be included in the tests. Therefore, for more accurate evaluation of the usefulness of the MALDI-TOF MS techniques and the database, re-identification of the tested 129 isolates using other methods such as *S. suis*-specific *recN* PCR (see below) and reassessment of the MALDI-TOF MS results using “authentic *S. suis*” will be needed.

### 4.2. Novel Methods for the Precise Identification and Detection of S. suis

Recently, Ishida et al. [47] considered the recent reclassification of this bacterium and developed a novel PCR assay for detecting *S. suis* strains. For this PCR assay, they selected *recN* as the target and designed two primers to detect only the serotype reference strains of authentic *S. suis* (i.e., serotype 1−19, 21, 23−25, 27−31, and 1/2 reference strains). As expected, under optimized conditions, the novel PCR (*recN* PCR) assay detected these serotype reference strains successfully, whereas no product was generated from the serotype 20, 22, 26, and 32–34 reference strains. Using *recN* PCR, a specific PCR product was also amplified from all 133 *S. suis* isolates of serotypes 1−5, 7−9, 11, 12, 15, 16, 25, and 31 tested; however, no amplicon was generated from any of the 16 isolates identified as *S. suis* serotypes 20, 22, and 33. Furthermore, this assay did not generate any specific amplicons from any other bacterial strains tested, including *S. gallinaceus* and *S. ovis* type strains, which displayed positive reactions using *gdh* PCR [47]. These findings suggest that the novel *recN* PCR assay is capable of distinguishing authentic *S. suis* strains from those of other species including *S. suis*-like strains. In 2015, a loop-mediated isothermal amplification (LAMP) method targeting *recN* of *S. suis* was reported and revealed to be useful for detecting *S. suis* from raw pork meat [68]. As these novel PCR and LAMP assays become more popular, the diagnosis of *S. suis* infections will become more accurate, and our understanding of the epidemiology of this important zoonosis will improve.

## 5. Current Topics on the Classification of *S. suis*-Like Strains

### 5.1. Whole-Genome Sequencing-Based Taxonomic Analyses in Bacteria

As described in an aforementioned section (Section 3.1), DDH remains the gold standard for the definitive assignment of a bacterial strain to a species. However, the results of DDH cannot be cumulated in databases, and this is a major drawback of this method in the bioinformatics era [69]. Therefore, there has been a continuous demand for an alternative genotype-based standard to replace DDH values [28,69]. Since the late 2000s, cost-effective and high-throughput DNA sequencing technologies have made whole-genome sequencing of bacterial strains more widely accessible, and direct comparisons of whole-genome sequences between strains are currently and readily applicable to bacterial taxonomy [70]. Average nucleotide identity (ANI) based on computational comparisons of two genome sequences is one of the similarity indices correlated with DDH values [71]. ANI is a mean of the similarity values of the total genomic sequence shared between two strains [71,72], and it has been most widely used as a possible next-generation gold standard for species delineation [30,69,71,72]. At present, it is accepted that ANI values of 95%–96% correspond to a DDH value of 70%, and they can be used as a cut-off point for a bacterial species boundary [69,72].

### 5.2. Streptococcus parasuis and Divergent S. suis Strains

DDH experiments performed by Tien et al. [20] indicated that the reference strains of serotypes 20, 22, and 26 belong to a single species taxonomically distinct from *S. suis*. To clarify the taxonomic position of these strains, Nomoto et al. [73] analyzed whole-genome sequences of the serotype 20, 22, and 26 reference strains and five additional *Streptococcus* strains that reacted with specific antisera of these serotypes and demonstrated that the ANI values among these eight strains were higher than the cut-off value for bacterial species (95.3%–99.9%), whereas the ANI values among the eight strains and strains belonging to the species *S. suis* (88.1%–89.0%) were much lower than the proposed cut-off value [73]. On the basis of these results and the results of additional phylogenetic and phenotypic analyses, the research group formally proposed these strains as the novel species *Streptococcus parasuis* [73].

On the contrary, Baig et al. [21] revealed nine “divergent *S. suis* strains” that were distinct from other “normal *S. suis* isolates” according to whole-genome sequence-based phylogeny on 390 *S. suis* strains, including 375 isolates identified using the API ID 32 Strep system. These divergent *S. suis* strains were classified into three genomic clades (Clades 1–3). Among the three clades, Clade 3 included the *S. suis* reference strains of serotypes 20, 22, and 26 [21] that were proposed as *S. parasuis* by Nomoto et al. [73]. In this study, although all of the divergent *S. suis* strains were distinguished from normal *S. suis* isolates via phylogenetic analysis of the *recN* sequences, not all of the divergent *S. suis* strains could be discriminated from normal *S. suis* isolates by phylogenetic comparisons based on the 16S rRNA, *sodA*, and *cpn60* sequences [20]. In addition, three divergent *S. suis* strains belonging to Clade 2 possessed CPS synthesis genes (*cps* genes) of *S. suis* serotype 4 reference strains [21,74]. In fact, two of these strains were serotyped as serotype 4 [21]. The phylogenies of 132 core genes shared between the nine divergent *S. suis* strains and strains of other streptococcal species demonstrated that these divergent *S. suis* strains were more closely related to normal *S. suis* isolates than to other streptococcal species [21]. From these results, the authors argued that reclassification of the divergent *S. suis* strains would be premature and that they should remain classified as divergent *S. suis* strains [21]. However, when attention is focused on only the Clade 3 strains, all of the strains, including *S. parasuis* strains (reference strains of serotypes 20, 22, and 26), were clustered in the same clade in any of the phylogenetic trees shown in the study, and the clade was apparently separated from the clades of normal *S. suis* isolates and the other divergent *S. suis* strains (Clade 1 and 2 strains) [21]. Therefore, some of their data may support the reclassification of serotype 20, 22, and 26 reference strains by Nomoto et al. [73].

Recently, Okura et al. identified many *S. suis*-like strains isolated from diseased and healthy ruminants (cattle, sheep, and a goat) that should be assigned to a novel species (unpublished data). These *S. suis*-like strains were suggested to be the same species as the serotype 33 reference strain isolated from a diseased lamb by ANI analyses and 16S rRNA gene sequences (unpublished data). Interestingly, to the best of our knowledge, similar *S. suis*-like strains have not been isolated from pigs, suggesting this novel species prefers ruminants rather than pigs. These *S. suis*-like strains sometimes cause confusion in the diagnosis of streptococcal diseases of ruminants because they are identified as *S. suis* by routine methods for identifying *Streptococcus* species. Therefore, a formal proposal of a novel species name for these strains and the development of a novel identification method of this species would contribute to the avoidance of confusion in veterinary diagnostic laboratories.

The *S. suis*-like strains identified to date are summarized in Table 2. Isolation of *S. suis*-like strains from humans has not been reported hitherto. Although we cannot hope to determine at this stage whether all the human clinical isolates reported as *S. suis* were authentic *S. suis* or not, it is noteworthy that most of the human clinical isolates reported so far were serotype 2, and no serotypes 20, 22, 26, and 32–34 strains have been isolated from humans [2]. In addition, most human clinical isolates analyzed were classified into limited clonal complexes (CC1, CC20, CC25, CC28, CC104, CC221/234, and CC233/379) by MLST [2,17,75,76,77,78,79], and as far as our and other groups analyzed by the whole-genome-based phylogenies, CC1, CC25, CC28, and CC104 strains from humans were grouped together with other authentic (normal) *S. suis* ([21] and unpublished observation).

## 6. Conclusions

*S. suis*, an important zoonotic agent, is composed of phenotypically and genetically diverse strains. Recently, several studies indicated the presence of “*S. suis*-like strains” that were revealed to be non-*S. suis* strains by taxonomic analyses based on genetic methods despite being previously identified as *S. suis* by biochemical tests and, in some cases, by clinical symptoms. Among the taxonomic analyses, *recN* sequence-based phylogeny is, in particular, an easy and very powerful tool for the discrimination of “authentic *S. suis*” from these *S. suis*-like strains and other *Streptococcus* species. Therefore, PCR and LAMP assays designed on the basis of *recN* sequences will be useful methods for more precise identification and detection of *S. suis*.

Classification of some *S. suis*-like strains, such as the “divergent *S. suis* strains”, is currently controversial. Little is known about these *S. suis*-like strains, including their potential virulence, association with diseases, host specificity, ecological importance, and distinctive phenotypic or genetic properties useful for discriminating the strains from authentic *S. suis*. More extensive studies using a number of *S. suis* and *S. suis*-like strains will provide additional insights into the classification of these strains and make the species boundaries clear.

## Figures and Tables

**Table 1:** Molecular biological approaches developed to aid in the serotyping of *S. suis*.

Method	Detecting Serotypes and Descriptions ^a^	Year	Reference
PCR (3 assays)	Assay 1: Serotypes 1 and 14;	1999	[49]
	Assay 2: Serotypes 2 and 1/2;		
	Assay 3: Serotype 9		
PCR	Serotype 7	1999	[50]
Multiplex-PCR	Serotypes 1, 2, 1/2, 7, 9, and 14	2002	[51]
	*epf* (a virulence-associated marker of *S. suis*) is also detected		
Multiplex-PCR	Serotypes 2 and 1/2	2004	[52]
	*S. suis*-specific sequence of the 16S rRNA gene is also detected		
Multiplex-PCR	Serotypes 1, 2, 1/2, 7, 9, and 14	2006	[53]
	*epf, sly*, *mrp*, *arcA* (virulence-associated markers of *S. suis*), and *S. suis*-specific sequence of *gdh* are also detected		
PCR	Serotype 16	2011	[54]
Real-time PCR	Serotypes 2 and 1/2	2011	[55]
PCR (8 assays)	Assay 1: Serotype 3; Assay 2: Serotype 4;	2012	[56]
	Assay 3: Serotype 5; Assay 4: Serotype 8;		
	Assay 5: Serotype 10; Assay 6: Serotype 19;		
	Assay 7: Serotype 23; Assay 8: Serotype 25;		
Multiplex-PCR (2 reaction sets)	15 serotypes (serotypes 1−5, 7−10, 14, 16, 19, 23, 25, and 1/2)	2012	[57]
	Reaction 1: Serotypes 1, 2, 1/2, 3, 4, 7, 9, 14, and 16		
	Reaction 2: Serotypes 5, 8, 10, 19, 23, and 25		
	In both reactions, the *S. suis*-specific sequence of the *gdh* gene is also detected		
Multiplex-PCR (4 reaction sets)	33 serotypes (serotypes 1−31, 33, and 1/2) and variant serotype 21/29	2013	[15]
	Reaction 1: Serotypes 1−10, 14, and 1/2		
	Reaction 2: Serotypes 11−21		
	Reaction 3: Serotypes 22−33		
	Reaction 4: Serotype 21/29		
	In all reactions, the *S. suis*-specific sequence of the *thrA* gene is also detected		
LAMP	Serotypes 2 and 1/2	2013	[58]
Multiplex-PCR (2-step assay)	35 serotypes (serotypes 1−34 and 1/2)	2014	[48]
	Step 1: classified into 7 groups (Group I−VII)		
	Group I: serotypes 3, 13, and 18		
	Group II: serotypes 1, 2, 1/2, 6, 14, 16, and 27		
	Group III: serotypes 21, 28, 29, and 30		
	Group IV: serotypes 4, 5, 7, 17, 19, and 23		
	Group V: serotypes 8, 15, 20, 22, and 25		
	Group VI: serotypes 9, 10, 11, 12, 24, 26, and 33		
	Group VII: serotypes 31, 32, and 34		
	Step 2: classified into respective serotypes of each group		
	In all reactions, universally shared sequences of the 16S rRNA gene are also detected		
Multiplex-PCR (4 reaction sets)	29 serotypes (serotypes 1−19, 21, 23−25, 27−31, 33, and 1/2)	2014	[59]
	Reaction 1: Serotypes 1, 2, 1/2, 3, 7, 9, 11, 14, and 16		
	Reaction 2: Serotypes 4, 5, 8, 12, 18, 19, 24, and 25		
	Reaction 3: Serotypes 6, 10, 13, 15, 17, 23, and 31		
	Reaction 4: Serotypes 21, 27, 28, 29, and 30		
	In all reactions, the *S. suis*-specific sequence of the *gdh* gene is also detected		
PCR	Serotype Chz	2015	[14]
luminex xTAG^®^ assay^™^	33 serotypes (serotypes 1−31, 33, and 1/2)	2015	[60]

a. *epf*, encoding an extracellular factor; *sly*, encoding suilysin; *mrp*, encoding muramidase-released protein; *arcA*, encoding arginine deiminase; *gdh*, encoding glutamate dehydrogenase; *thrA*, encoding aspartokinase/homoserine dehydrogenase I; LAMP, loop-mediated isothermal amplification.

**Table 2:** “*S. suis*-like strains” reported or identified in previous studies.

Strains	Serotype ^a^	Source	Descriptions ^b^	Reference
EA1172.91	32	Diseased pig (septicemia)	Serotype reference strains Considered to be *S. orisratti* by *cpn60* analysis	[19]
92-2742	34	Diseased pig (aborted fetus)		
EA1832.92	33	Diseased lamb (arthritis)	Serotype reference strain Shown to be a non-*S. suis* strain by DDH	[20]
86-5192	20	Diseased pig (unknown)	Serotype reference strains Shown to be non-*S. suis* strains and the same species by DDH and ANI Reclassified as *S. parasuis* [73] or considered to be divergent *S. suis* strains (Classified into Clade 3 on the basis of the whole-genome-based phylogeny [21])	[20,21,73]
88-1861	22	Diseased calf (unknown)		
89-4109-1	26	Diseased pig (unknown)		
SUT-286	20	Healthy pigs (saliva)	*S. parasuis* strains (SUT-286 is the type strain) Shown to be the same species by ANI	[73]
SUT-380	22			
SUT-319, 328	20/22			
SUT-7	22/26			
LSS7	UT	Healthy pigs	Divergent *S. suis* strain Classified into Clade 1 by the whole genome-based phylogeny	[21]
SS007	4	Diseased pig (systemic-brain infection)	Divergent *S. suis* strains *cps* losus of LSS6 was similar to *cps4* locus Classified into Clade 2 by the whole genome-based phylogeny	[21]
LSS19	4	Healthy pig		
LSS6	UT	Healthy pig		
SS1003	22	Diseased pig (respiratory infection)		
LSS17	UT	Healthy pig	Divergent *S. suis* strain Classified into Clade 3 by the whole genome-based phylogeny	[21]
SUT-283	20	Healthy pig	*recN* PCR negative but *gdh* PCR positive strains	[47]
FUT-29	20	Pork		
GUT-182	22	Diseased pig (endocarditis)		
GUT-183−193 (11 strains)	33	Diseased calves (endocarditis)		
More than 70 isolates	33 and UT	Diseased cattle, sheep, and a goat (endocarditis, arthritis, and pneumonia) Healthy cattle (tonsil and nasal cavity)	*recN* PCR negative but *gdh* PCR positive strains Twenty of them analyzed by whole genome sequencing were shown to be the same species with serotype 33 reference strain by ANI	Unpublished

^a^ UT, unserotypable; ^b^ DDH, DNA–DNA hybridization; ANI, average nucleotide identity.

## Abbreviations

The following abbreviations are used in this manuscript:

CPS	capsular polysaccharide
ST	Sequence type
DNA	deoxyribonucleic acid
DDH	DNA–DNA hybridization
16S rRNA	16 Svedberg units ribosomal-ribonucleic acid
API	analytical profile index
PCR	polymerase chain reaction
MALDI-TOF MS	Matrix-assisted laser desorption ionization time-of-flight mass spectrum
LAMP	loop-mediated isothermal amplification
ANI	average nucleotide identity
UT	unserotypable
